# Alpha-lipoic acid, apocynin or probiotics influence glutathione status and selected inflammatory parameters in C57/BL6 mice when combined with a low-fat diet

**DOI:** 10.1007/s43440-023-00527-8

**Published:** 2023-09-21

**Authors:** Paulina Kleniewska, Rafał Pawliczak

**Affiliations:** Department of Immunopathology, Faculty of Medicine, Medical University of Lodz, Żeligowskiego 7/9 (Bldg 2 Rm 177), 90-752 Łódź, Poland

**Keywords:** Reactive oxygen species, Antioxidants, Low-fat diet, High-fat diet, Oxidative stress

## Abstract

**Background:**

The aim of the study was to determine the potential of a low-fat diet (LFD) to protect against oxidative and inflammatory damage in the course of asthma and obesity when combined with antioxidants (alpha-lipoic acid–ALA, apocynin–APO) or a probiotic (P) (*Lactobacillus casei*).

**Methods:**

The experiments were carried out on ten groups of male C57/BL6 mice that were fed standard fat (SFD), low-fat (LFD), or high-fat (HFD) diets. Ovalbumin (OVA, administered subcutaneously and by inhalation) was used to sensitize the animals. IL-1α, IL-10, eotaxin-1, leptin, and TNF-α concentrations were examined in blood, while total glutathione (GSHt), reduced glutathione (GSH), oxidized glutathione (GSSG) and –SH groups were measured in lung homogenates.

**Results:**

LFD in combination with the analyzed compounds (APO, P, ALA) significantly decreased the concentration of IL-1α compared to the OVA + HFD group (*p* < 0.01; *p* = 0.025; *p* = 0.002, respectively). Similarly, the treated mice demonstrated lower eotaxin-1 concentrations compared to the HFD group (*p* < 0.001). Moreover, supplementation of LFD with probiotics significantly increased the concentration of IL-10 vs. controls (*p* < 0.001) and vs. untreated OVA-sensitized and challenged/obese mice (*p* < 0.001). Animals administered APO/ALA with LFD displayed a significant decrease in TNF-α concentration compared to OVA + HFD mice (*p* = 0.013; *p* = 0.002 respectively). Those treated with ALA displayed significantly improved GSH levels (*p* = 0.035) compared to OVA + HFD mice.

**Conclusions:**

Supplementation of the tested compounds with LFD appears to have a positive influence on the glutathione redox status of pulmonary tissues and selected inflammatory parameters in mouse blood.

**Supplementary Information:**

The online version contains supplementary material available at 10.1007/s43440-023-00527-8.

## Introduction

According to data published by the World Health Organization (WHO), obesity has nearly tripled worldwide since 1975. Obesity and asthma frequently coexist [1]. Recent studies indicate that in obese people, the development of bronchial asthma is closely associated with oxidative stress (OS), caused by an imbalance between the production and elimination of reactive oxygen species (ROS). Fortunately, cells have a variety of defense mechanisms to ameliorate the harmful effects of ROS. These defenses employ both enzymatic tools, such as catalase (CAT), superoxide dismutase (SOD), and glutathione peroxidase (GPx), and non-enzymatic ones, such as vitamin C and E, reduced glutathione (GSH), albumin, and bilirubin. However, a fall in cellular antioxidant production can result in OS [2]. This can be exacerbated by an increase in ROS derived from exogenous sources, such as air pollution, UV, ionizing radiation, tobacco smoke, pesticides, industrial solvents, and food preservatives, and endogenous ones, such as inflammatory processes, and fatty acid oxidation, the mitochondrial respiratory chain, cytochrome P450, or lipoxygenase [3].

One of the enzymes that produce superoxide anions is NADPH oxidase. One of its best-known natural inhibitors is apocynin (acetovanillone) [4–7]. Apocynin (APO) was first extracted from the roots of *Apocynum cannabinum*, then from the root extract of the medicinal herb *Picrorhiza kurroa*. This compound is known to inhibit NADPH oxidase activity via blockage of the p47phox subunit, thus lowering ROS concentration and reducing OS. In addition, APO itself is also characterized by selective action, low toxicity, and no side effects, which further increases its therapeutic attractiveness.

Lipoic acid (LA) or alpha lipoic acid (ALA) is absorbed mainly in the form of liposin, which is hydrolyzed to LA in the blood by lipoamidase, with the simultaneous release of liponate. The LA is then transported to the tissues, where it is reduced to biologically active dihydrolipoic acid (DHLA) by lipoamide dehydrogenase. Thanks to the ability of DHLA to reduce the oxidized forms of other antioxidants, including glutathione disulfide (GSSG), LA is commonly referred to as a “universal antioxidant”. It has been shown that lipoic acid supplementation reduces the concentration of malondialdehyde (MDA) and increases GSH/GSSG ratio, total antioxidant capacity (TAC), and GSH and total glutathione (GSHt) in RBC lysate [8]. Treatment with LA may effectively reduce the elevated concentration of MDA in the lungs induced by amiodarone and positively influence the content of GSH in the lungs and TAC in the serum [9].

Both compounds (lipoic acid and apocynin) are highly effective antioxidants [8–11].

Probiotic strains have been reported to produce antioxidants and scavenge hydroxyl radicals/superoxide anions. The most popular bacterial strains include *Lactobacillus* and *Bifidobacterium* [12, 13]. Probiotics inhibit the inflammatory process by lowering the activity of nuclear factor kappa B (NF-κB), resulting in a decrease in tumor necrosis factor-alpha (TNF-α), interleukin 1, 6 (IL-1, IL-6), and C-reactive protein (CRP) production. It is worth noting that probiotics stimulate the activity of GSH and CAT [14], reduce the production of ROS in the intestines, and remove peroxides, heavy metals, and hydroxyl radicals [15–17]. The administration of probiotics protects against the development of oxidative stress [14–16] in the course of obesity-associated asthma.

This work investigates the influence of LFD administration, alone or in combination with ALA, APO, or *Lactobacillus casei*, on oxidative/inflammatory processes occurring in the course of asthma and/or obesity. The antioxidant and anti-inflammatory properties of the tested compounds create many therapeutic perspectives. However, further studies are needed to transfer our findings into clinical practice.

## Materials and methods

### Chemicals

Acetovanillone ≥ 98% (Item No. W508454), ( ±)-α-lipoic acid synthetic (Item No. T5625 ≥ 99%, powder), OVA-albumin from chicken egg white (Item No. A5503; lyophilized powder, ≥ 98%, grade V), β-NADPH (β-nicotinamide adenine dinucleotide phosphate, Item No. N3886), Na_2_HPO_4_ (Item No. 567550), triethanolamine hydrochloride (TEA, Item No. T1502), 5,5′-dithio-bis (2-nitrobenzoic acid, DTNB, Item No. D8130), 2-vinylpyridine (Item No. 132292) and glutathione reductase (GR, Item No. G3664) were purchased from Sigma Chemical Co. (St. Louis, MO, USA). Trichloroacetic acid (TCA, Item No. 577970115) and 5-sulfosalicylic acid hydrate (5-SSA, Item No. 575640115) were purchased from Pol-Aura Sp. z o. o. (Lodz, Poland). All other reagents were obtained from R&D Systems Inc. (Minneapolis, MN, USA) and Biorbyt Ltd. (Cambridge, Cambs, UK).

### Animals and diets

**Fig. 1:**
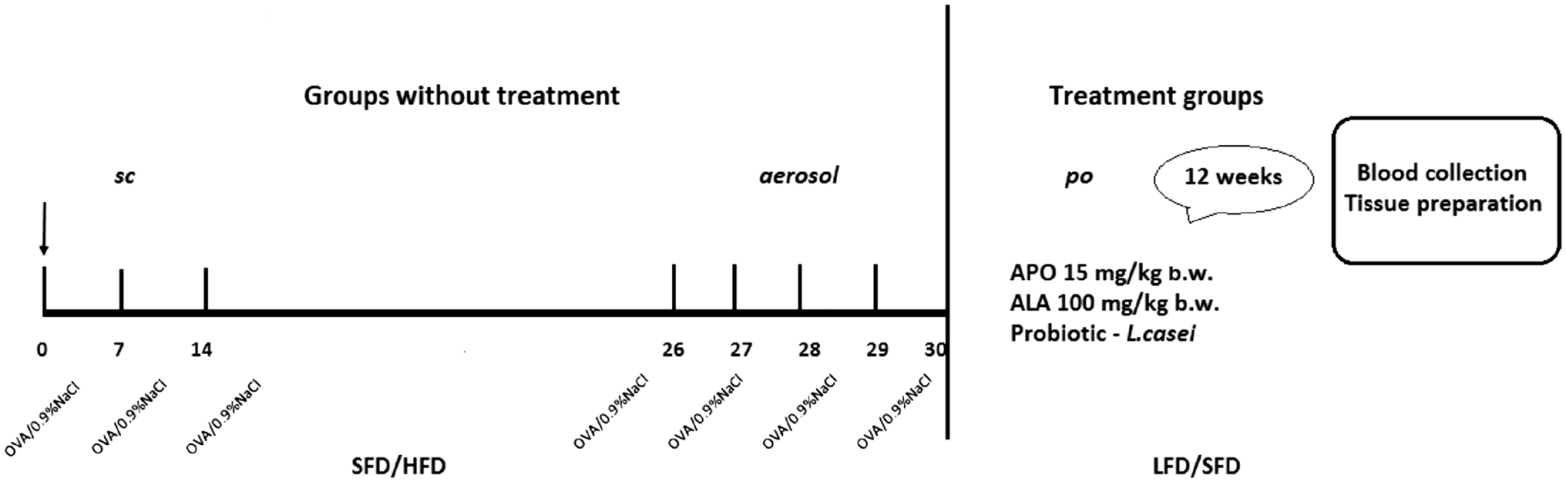
Experimental design

The paper was based on murine models of chronic asthma created via ovalbumin challenge (100 µg + 1% solution) by subcutaneous injections on days 0, 7, and 14 + inhalations, or of obesity, created by feeding with an HFD for 12 weeks [18, 19] (Fig. 1). All experiments were carried out on adult male C57/BL6 mice. The animals were randomly divided into ten groups of seven individuals each. All were kept under standard laboratory lighting and temperature conditions (12 h artificial lighting/12 h dark; room temperature) with free access to appropriate lab chow and tap water.

Mice from the untreated groups were fed for 12 weeks with either a standard fat diet (SFD) consisting of 71.3 g carbohydrate, 20.3 g protein, and 4.6 g fat (Group 1–control; Group 2–OVA), or a high-fat diet (HFD) consisting of 42.1 g carbohydrate, 25.1 g protein and 28.3 g fat (Group 3–HFD; Group 4–OVA + HFD).

The treatment groups received an appropriate diet along with antioxidants or probiotics. For the next 12 weeks, they either received a low-fat diet (LFD) consisting of 74.5 g carbohydrate, 19.9 g protein, and 1.9 g fat (alone or with APO, ALA, or a probiotic–P) or SFD in combination with the tested compounds. Group 5 received APO *po* (15 mg/kg) plus SFD; Group 6 received LFD; Group 7 was treated with LFD plus APO *po* (15 mg/kg); Group 8 received LFD with probiotic (*L. casei po*); Group 9 received LFD plus alpha-lipoic acid *po* (100 mg/kg); Group 10 was treated with *L. casei po* plus SFD. The doses of all tested compounds were based on body weight.

All test procedures were approved by the Medical University of Lodz Ethics Committee (No. 26/ŁB59/2017).

### Biochemical determinations

The levels of eotaxin-1, IL-1α, IL-10, TNF-α, and leptin in mouse blood were estimated by ELISA with commercially available materials (R&D Systems Inc. and Biorbyt Ltd.). The measurements were performed according to the manufacturer’s instructions (detailed description in Supplementary file).

### Measurement of glutathione concentrations in the lung homogenates

Weighted pieces of tissue were homogenized in an ice-cold solution of 5% 5-SSA and centrifuged (10,000 × g, 10 min, 4 °C). The total glutathione content of the supernatant was measured in a 1000 µl cuvette containing 50 µl of the sample and 700 µl of 0.2 mM NADPH, 100 µl of 0.6 mM DTNB + 150 µl of H_2_O. Following this, the cuvette was incubated for five minutes at 37 °C. 0.6 U/l of GR was then added. The reaction kinetics were followed for five minutes by monitoring the increase in absorbance.

Glutathione disulfide level was measured in the supernatant using the same method after optimization of pH to 6–7 with 1 M TEA and derivatization of endogenous GSH with 2-vinylpyridine (v:v). Absorbance was read at 412 nm. The reduced glutathione level in the supernatant was calculated as the difference between total glutathione and glutathione disulfide. The results were expressed in µmol.

### Measurement of –SH groups concentrations in the lung homogenates

The concentration of sulfhydryl groups was determined spectrophotometrically according to Ellman (1970) using a Perkin-Elmer Lambda 25 spectrophotometer. Weighted pieces of tissue were homogenized in an ice-cold solution of 6% TCA and centrifuged (10,000 × g, 10 min, 4 °C). Following this, 500 μl of the sample, 500 μl of 0.3 M Na_2_HPO_4_, and 500 μl of 0.04% Ellman reagent (freshly dissolved in a solution of 10% sodium citrate) were added to the measuring cuvette. Absorbance was read at 412 nm. The results were expressed in µmol.

### Statistical analysis

Differences between groups were assessed using one-way ANOVA combined with the following post hoc tests: Dunnett’s method (multiple comparisons versus control group), Tukey’s multiple-range test, Duncan’s method (all pairwise multiple comparison procedures) or Dunn’s method (following rejection of Kruskal–Wallis test). All data were presented as mean ± standard error of the mean (SEM). Differences were considered significant at a *p*-value less than 0.05.

## Results

### Evaluation of eotaxin-1, IL-1α, IL-10, TNF-α and leptin concentrations

**Fig. 2:**
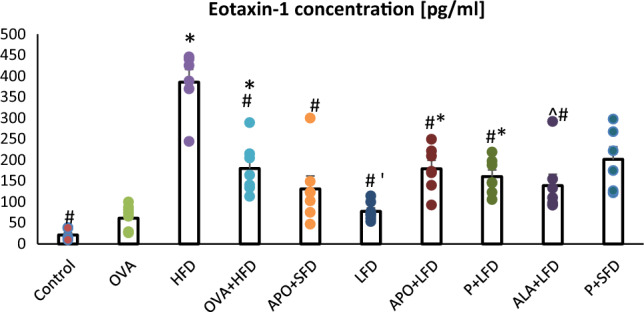
The concentration of eotaxin-1 in the plasma of C57/BL6 mice. Control–saline (100 µg subcutaneously + 0.9% of NaCl by inhalation) with SFD; OVA–ovalbumin (100 µg subcutaneously + 1% solution by inhalation) with SFD; HFD–saline (100 µg subcutaneously + 0.9% of NaCl by inhalation) with HFD; OVA + HFD–ovalbumin (100 µg subcutaneously + 1% solution by inhalation) plus HFD. Groups with therapeutic intervention: APO + SFD–asthmatic and obese mice that received apocynin *po* (15 mg/kg b.w.) plus SFD; LFD–obese asthmatic animals that received LFD; APO + LFD–obese asthmatic mice treated with LFD plus apocynin *po* (15 mg/kg b.w.); P + LFD—obese asthmatic animals that received LFD with probiotic (*L. casei*); ALA + LFD–obese asthmatic animals that received LFD plus alpha-lipoic acid *po* (100 mg/kg b.w.); P + SFD–obese asthmatic mice treated with probiotic (*L. casei*) plus SFD. Data are shown as mean ± SEM; one-way ANOVA; *p < 0.001; ^&^*p* = 0.007; ^*p* = 0.003 vs. control (Dunnett’s post hoc test); ^#^*p* < 0.001 vs. HFD; ‘*p* = 0.038 vs. OVA + HFD (Tukey’s post hoc test); *n* = 7. *LFD*–low-fat diet; *HFD*–high-fat diet; *SFT*–standard fat diet

**Fig. 3:**
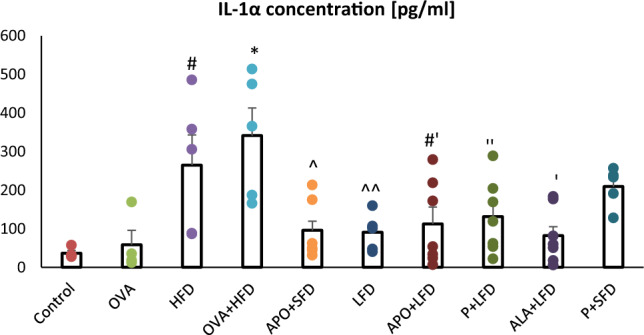
The concentration of IL-1α in the plasma of C57/BL6 mice. Control–saline (100 µg subcutaneously + 0.9% of NaCl by inhalation) with SFD; OVA–ovalbumin (100 µg subcutaneously + 1% solution by inhalation) with SFD; HFD–saline (100 µg subcutaneously + 0.9% of NaCl by inhalation) with HFD; OVA + HFD–ovalbumin (100 µg subcutaneously + 1% solution by inhalation) plus HFD. Groups with therapeutic intervention: APO + SFD–asthmatic and obese mice that received apocynin *po* (15 mg/kg b.w.) plus SFD; LFD–obese asthmatic animals that received LFD; APO + LFD–obese asthmatic mice treated with LFD plus apocynin *po* (15 mg/kg b.w.); P + LFD–obese asthmatic animals that received LFD with probiotic (*L. casei*); ALA + LFD–obese asthmatic animals that received LFD plus alpha-lipoic acid *po* (100 mg/kg b.w.); P + SFD–obese asthmatic mice treated with probiotic (*L. casei*) plus SFD. Data are shown as mean ± SEM; one-way ANOVA; *p < 0.001; ^#^*p* = 0.01 vs. control (Dunnett’s post hoc test); ^*p* = 0.007; ^^*p* = 0.009; ^#’^*p* < 0.01; ‘’*p* = 0.025; ‘*p* = 0.002 vs. OVA + HFD (Tukey’s post hoc test); *n* = 7. *LFD*–low-fat diet; *SFD*–standard fat diet; *HFD*–high-fat diet

**Fig. 4:**
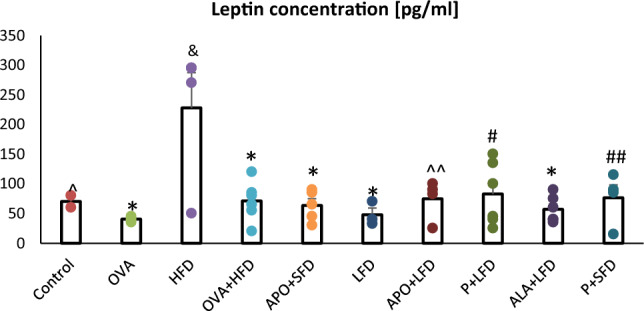
The concentration of leptin in the plasma of C57/BL6 mice. Control–saline (100 µg subcutaneously + 0.9% of NaCl by inhalation) with SFD; OVA–ovalbumin (100 µg subcutaneously + 1% solution by inhalation) with SFD; HFD–saline (100 µg subcutaneously + 0.9% of NaCl by inhalation) with HFD; OVA + HFD–ovalbumin (100 µg subcutaneously + 1% solution by inhalation) plus HFD. Groups with therapeutic intervention: APO + SFD–asthmatic and obese mice that received apocynin *po* (15 mg/kg b.w.) plus SFD; LFD–obese asthmatic animals that received LFD; APO + LFD–obese asthmatic mice treated with LFD plus apocynin *po* (15 mg/kg b.w.); P + LFD–obese asthmatic animals that received LFD with probiotic (*L. casei*); ALA + LFD–obese asthmatic animals that received LFD plus alpha-lipoic acid *po* (100 mg/kg b.w.); P + SFD—obese asthmatic mice treated with probiotic (*L. casei*) plus SFD. Data are shown as mean ± SEM; one-way ANOVA; ^&^*p* = 0.004 vs. control (Dunnett’s post hoc test); *p < 0.001; ^*p* = 0.02; ^^*p* = 0.003; ^#^*p* = 0.002; ^##^*p* = 0.004 vs. HFD (Tukey’s post hoc test); *n* = 7. *LFD*–low-fat diet; *HFD*–high-fat diet; *SFD*–standard fat diet.

In the HFD and OVA + HFD groups, the levels of eotaxin-1 (*p* < 0.001) and IL-1α (*p* = 0.01; *p* < 0.001, respectively) were significantly higher than control values (Figs. 2 and 3). The HFD group also demonstrated significantly higher blood leptin concentration than controls (*p* = 0.004; Fig. 4).

Administration of APO with SFD significantly decreased the concentration of eotaxin-1 as compared to the HFD group (*p* < 0.001). Significantly lower concentrations of IL-1α were observed in the LFD group (*p* = 0.009), APO + LFD group (*p* < 0.01), ALA + LFD group (*p* = 0.002), and P + LFD group (*p* = 0.025) compared to the OVA + HFD group. The administration of a low-fat diet, either alone or in combination with APO, P, or ALA, resulted in a significant decrease in eotaxin-1 concentration compared to the HFD group (*p* < 0.001). Lower levels of eotaxin and IL-1α were also observed in the P + SFD group; however, these changes were not significant (Figs. 2 and 3; one-way ANOVA, *F*_9_ = 19.68 and *F*_9_ = 4.75_,_ *p* < 0.001 respectively).

**Fig. 5:**
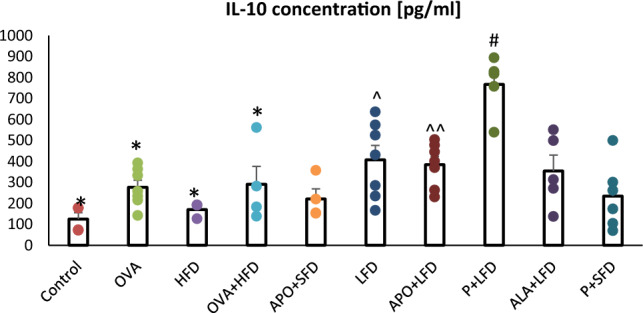
The concentration of IL-10 in the plasma of C57/BL6 mice. Control–saline (100 µg subcutaneously + 0.9% of NaCl by inhalation) with SFD; OVA–ovalbumin (100 µg subcutaneously + 1% solution by inhalation) with SFD; HFD–saline (100 µg subcutaneously + 0.9% of NaCl by inhalation) with HFD; OVA + HFD–ovalbumin (100 µg subcutaneously + 1% solution by inhalation) plus HFD. Groups with therapeutic intervention: APO + SFD–asthmatic and obese mice that received apocynin *po* (15 mg/kg b.w.) plus SFD; LFD–obese asthmatic animals that received LFD; APO + LFD–obese asthmatic mice treated with LFD plus apocynin *po* (15 mg/kg b.w.); P + LFD–obese asthmatic animals that received LFD with probiotic (*L. casei*); ALA + LFD—obese asthmatic animals that received LFD plus alpha-lipoic acid *po* (100 mg/kg b.w.); P + SFD–obese asthmatic mice treated with probiotic (*L. casei*) plus SFD. Data are shown as mean ± SEM; one-way ANOVA; ^#^*p* < 0.001; ^*p* = 0.013; ^^*p* = 0.026 vs. control (Dunnett’s post hoc test); *p < 0.001 vs. P + LFD (Tukey’s post hoc test); *n* = 7. *LFD*–low-fat diet; *HFD*–high-fat diet; *SFD*–standard fat diet

Supplementation of a low-fat diet with probiotics significantly increased the concentration of IL-10 (Fig. 5) in comparison with controls (*p* < 0.001) and the OVA- challenged/obese mice without treatment (one-way ANOVA, *F*_9_ = 8.38, *p* < 0.001).

A significant decrease in leptin concentrations was observed for LFD alone (*p* = 0.001) and in combination with APO (*p* = 0.003), probiotics (*p* = 0.002) or ALA (*p* < 0.001) compared with the HFD group. Leptin was also lower in the OVA group vs*.* control. However, no significant changes were observed. Moreover, significantly lower leptin levels were observed in groups fed SFD with APO (*p* < 0.001) and *L. casei* (*p* = 0.004) compared to the HFD group (Fig. 4; one-way ANOVA, *F*_9_ = 4.78,* p* < 0.001).

**Fig. 6:**
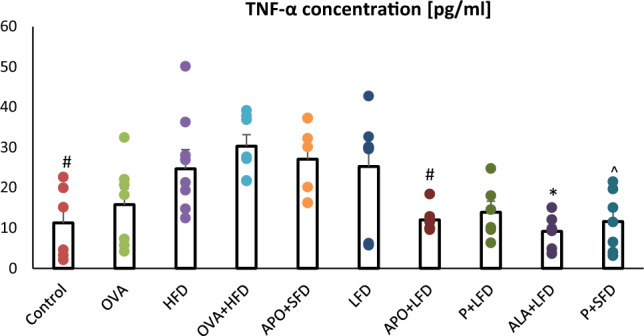
The concentration of TNF-α in the plasma of C57/BL6 mice. Control–saline (100 µg subcutaneously + 0.9% of NaCl by inhalation) with SFD; OVA–ovalbumin (100 µg subcutaneously + 1% solution by inhalation) with SFD; HFD–saline (100 µg subcutaneously + 0.9% of NaCl by inhalation) with HFD; OVA + HFD–ovalbumin (100 µg subcutaneously + 1% solution by inhalation) plus HFD. Groups with therapeutic intervention: APO + SFD–asthmatic and obese mice that received apocynin *po* (15 mg/kg b.w.) plus SFD; LFD–obese asthmatic animals that received LFD; APO + LFD—obese asthmatic mice treated with LFD plus apocynin *po* (15 mg/kg b.w.); P + LFD–obese asthmatic animals that received LFD with probiotic (*L. casei*); ALA + LFD–obese asthmatic animals that received LFD plus alpha-lipoic acid *po* (100 mg/kg b.w.); P + SFD–obese asthmatic mice treated with probiotic (*L. casei*) plus SFD. Data is shown as mean ± SEM; one-way ANOVA; ^#^*p* = 0.013; *p = 0.002; ^*p* = 0.010 vs. OVA + HFD (Tukey’s post hoc test); *n* = 7. *LFD*–low-fat diet; *HFD*–high-fat diet; *SFD*–standard fat diet

Animals administered APO/ALA with LFD displayed a significant decrease in TNF-α concentration compared to the OVA + HFD group (*p* = 0.013; *p* = 0.002, respectively). Those treated with probiotics displayed lower TNF-α concentrations (*p* = 0.010) than the obese asthmatic animals (Fig. 6; one-way ANOVA, *F*_9_ = 5.15, *p* < 0.001).

### Evaluation of GSHt, GSSG, and GSH concentrations and –SH group content

**Fig. 7:**
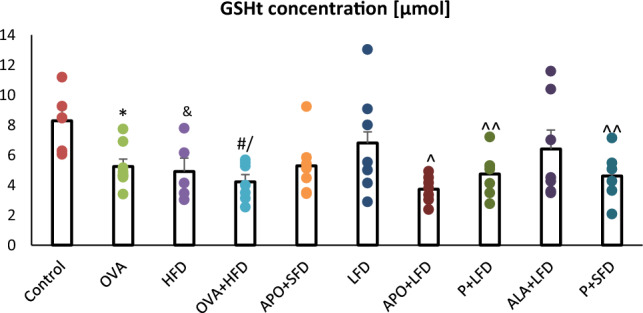
The concentration of total glutathione in the lung homogenates of C57/BL6 mice. Control–saline (100 µg subcutaneously + 0.9% of NaCl by inhalation) with SFD; OVA–ovalbumin (100 µg subcutaneously + 1% solution by inhalation) with SFD; HFD–saline (100 µg subcutaneously + 0.9% of NaCl by inhalation) with HFD; OVA + HFD–ovalbumin (100 µg subcutaneously + 1% solution by inhalation) plus HFD. Groups with therapeutic intervention: APO + SFD–asthmatic and obese mice that received apocynin *po* (15 mg/kg b.w.) plus SFD; LFD–obese asthmatic animals that received LFD; APO + LFD–obese asthmatic mice treated with LFD plus apocynin *po* (15 mg/kg b.w.); P + LFD–obese asthmatic animals that received LFD with probiotic (*L. casei*); ALA + LFD–obese asthmatic animals that received LFD plus alpha-lipoic acid *po* (100 mg/kg b.w.); P + SFD–obese asthmatic mice treated with probiotic (*L. casei*) plus SFD. Data is shown as mean ± SEM; one-way ANOVA; ^#^*p* = 0.008; ^*p* = 0.002; ^^*p* = 0.026 vs. control (Dunnett’s post hoc test); *p = 0.018; ^&^*p* = 0.022; /*p* = 0.003 vs. control (Duncan’s post hoc test); *n* = 7. *LFD*–low-fat diet; *HFD*–high-fat diet; *SFD*–standard fat diet

Significantly lower levels of GSHt were observed in the lungs of OVA-sensitized + challenged (*p* = 0.018), obese (*p* = 0.022), and obese asthmatic (*p* = 0.003) animals compared to the control group. Figure 7 shows that mice treated with LFD or ALA + LFD demonstrated higher levels of total glutathione in lung homogenates than in the OVA + HFD group. However, these changes were not significant (one-way ANOVA, *F*_9_ = 2.71, *p* = 0.011).

**Fig. 8:**
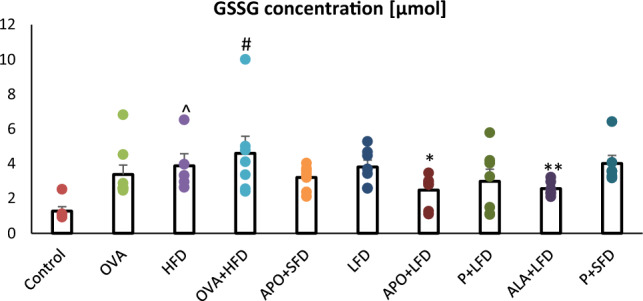
The concentration of oxidized glutathione in the lung homogenates of C57/BL6 mice. Control–saline (100 µg subcutaneously + 0.9% of NaCl by inhalation) with SFD; OVA–ovalbumin (100 µg subcutaneously + 1% solution by inhalation) with SFD; HFD–saline (100 µg subcutaneously + 0.9% of NaCl by inhalation) with HFD; OVA + HFD–ovalbumin (100 µg subcutaneously + 1% solution by inhalation) plus HFD. Groups with therapeutic intervention: APO + SFD–asthmatic and obese mice that received apocynin *po* (15 mg/kg b.w.) plus SFD; LFD–obese asthmatic animals that received LFD; APO + LFD–obese asthmatic mice treated with LFD plus apocynin *po* (15 mg/kg b.w.); P + LFD–obese asthmatic animals that received LFD with probiotic (*L. casei*); ALA + LFD–obese asthmatic animals that received LFD plus alpha-lipoic acid *po* (100 mg/kg b.w.); P + SFD–obese asthmatic mice treated with probiotic (*L. casei*) plus SFD. Data are shown as mean ± SEM; one-way ANOVA; ^#^*p* < 0.001; ^*p* = 0.024 vs. control (Dunnett’s post hoc test); *p < 0.018; **p = 0.028 vs. OVA + HFD (Duncan’s post hoc test); *n* = 7. *LFD*–low-fat diet; *HFD*–high-fat diet; *SFD*–standard fat diet

Significantly higher concentrations of GSSG were noted in the lungs of obese (*p* = 0.024) and obese asthmatic (*p* < 0.001) animals compared to controls. In addition, markedly lower levels of oxidized glutathione were observed in the lung homogenates of mice treated with LFD plus APO (*p* < 0.018) or plus ALA (*p* = 0.028) than in the OVA + HFD group (Fig. 8). In the SFD + APO group and SFD + P group, GSSG concentration also fell insignificantly (one-way ANOVA, *F*_9_ = 2.88, *p* = 0.007).

**Fig. 9:**
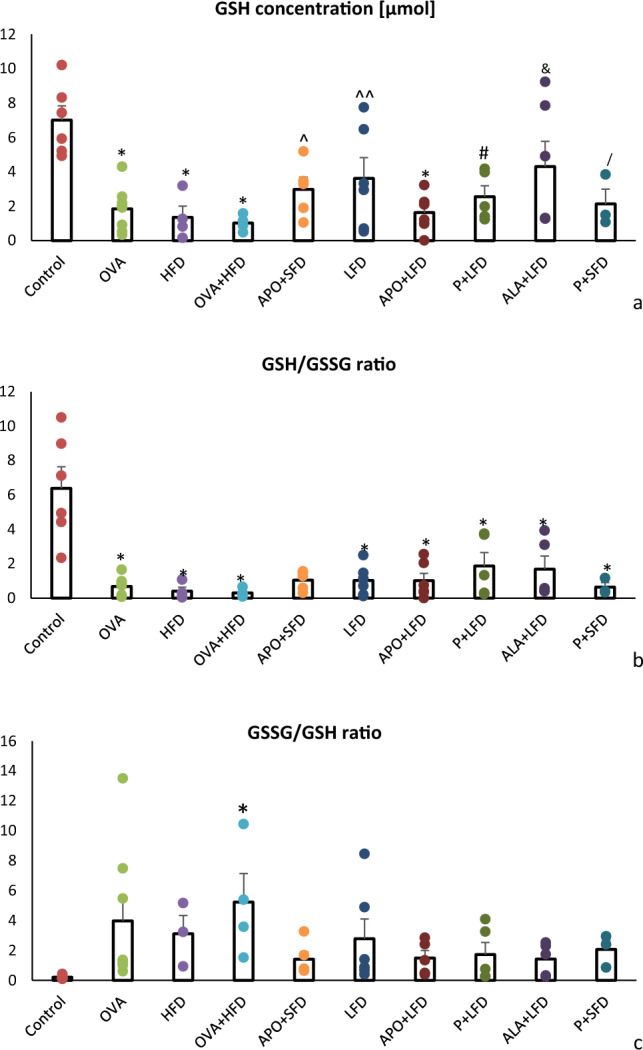
The concentration of reduced glutathione in the lung homogenates of C57/BL6 mice (**a**), changes in the reduced to oxidized glutathione ratio (**b**), changes in the oxidized to reduced glutathione ratio (**c**). Control–saline (100 µg subcutaneously + 0.9% of NaCl by inhalation) with SFD; OVA–ovalbumin (100 µg subcutaneously + 1% solution by inhalation) with SFD; HFD–saline (100 µg subcutaneously + 0.9% of NaCl by inhalation) with HFD; OVA + HFD–ovalbumin (100 µg subcutaneously + 1% solution by inhalation) plus HFD. Groups with therapeutic intervention: APO + SFD—asthmatic and obese mice that received apocynin *po* (15 mg/kg b.w.) plus SFD; LFD–obese asthmatic animals that received LFD; APO + LFD–obese asthmatic mice treated with LFD plus apocynin *po* (15 mg/kg b.w.); P + LFD–obese asthmatic animals that received LFD with probiotic (*L. casei)*; ALA + LFD–obese asthmatic animals that received LFD plus alpha-lipoic acid *po* (100 mg/kg b.w.); P + SFD–obese asthmatic mice treated with probiotic (*L. casei)* plus SFD. Data is shown as mean ± SEM; one-way ANOVA **a** *p < 0.001; ^*p* = 0.015; ^^*p* = 0.041; ^#^*p* = 0.006; /*p* = 0.011 vs. control (Dunnett’s test); ^&^*p* = 0.035 vs. OVA + HFD (Duncan’s test); **b** *p < 0.001 vs. control (Dunnett’s and Tukey’s post hoc tests); **c** *p < 0.05 vs. control (Dunn’s post hoc test); *n* = 7. *LFD*–low-fat diet; *HFD*–high-fat diet; *SFD*–standard fat diet

GSH levels were significantly lower in the lungs of OVA-sensitized + challenged, obese, and obese asthmatic animals than those of the control group (*p* < 0.001). ALA + LFD treatment significantly improved levels of GSH (*p* = 0.035) compared to OVA + HFD mice (Fig. 9 a; one-way ANOVA, *F*_9_ = 4.37_,_ *p* < 0.001).

The ALA + LFD group and P + LFD group demonstrated a higher GSH/GSSG ratio than the OVA + HFD group. The obese asthmatic animals demonstrated a higher GSSG/GSH ratio than controls (*p* < 0.05). The GSSG/GSH ratios in the APO + LFD, ALA + LFD, and P + LFD groups were insignificantly lower (*p* > 0.05) compared with obese asthmatic animals. These changes are presented in more detail in Fig. 9 (b and c; one-way ANOVA, *F*_9_ = 9.18, *p* < 0.001 and *F*_9_ = 1.56, *p* = 0.160 respectively).

**Fig. 10:**
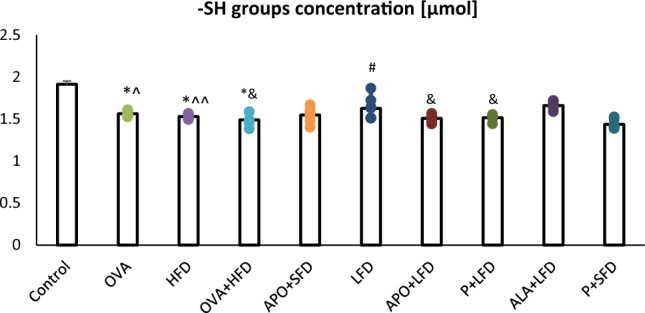
The concentration of –SH groups in the lung homogenates of C57/BL6 mice. Control–saline (100 µg subcutaneously + 0.9% of NaCl by inhalation) with SFD; OVA–ovalbumin (100 µg subcutaneously + 1% solution by inhalation) with SFD; HFD–saline (100 µg subcutaneously + 0.9% of NaCl by inhalation) with HFD; OVA + HFD–ovalbumin (100 µg subcutaneously + 1% solution by inhalation) plus HFD. Groups with therapeutic intervention: APO + SFD–asthmatic and obese mice that received apocynin *po* (15 mg/kg b.w.) plus SFD; LFD–obese asthmatic animals that received LFD; APO + LFD–obese asthmatic mice treated with LFD plus apocynin *po* (15 mg/kg b.w.); P + LFD–obese asthmatic animals that received LFD with probiotic (*L. casei)*; ALA + LFD–obese asthmatic animals that received LFD plus alpha-lipoic acid *po* (100 mg/kg b.w.); P + SFD–obese asthmatic mice treated with probiotic (*L. casei)* plus SFD. Data is shown as mean ± SEM, one-way ANOVA; *p < 0.001 vs. control; ^*p* = 0.013; ^^*p* = 0.005; ^&^*p* < 0.001 vs. ALA + LFD; ^#^*p* = 0.002 vs. OVA + HFD (Duncan’s post hoc test); *n* = 7. *LFD*–low-fat diet; *HFD–*high-fat diet; *SFD*–standard fat diet

The level of –SH groups was significantly decreased in the OVA group, HFD group, and OVA + HFD group when compared to controls (*p* < 0.001) (Fig. 10). The LFD group demonstrated higher –SH content (*p* = 0.002) than the OVA + HFD group. A significant increase in lung–SH groups was observed in the ALA + LFD group in comparison to the OVA (*p* = 0.013), HFD (*p* = 0.005), and OVA + HFD (*p* < 0.001) groups (one-way ANOVA, *F*_9_ = 21.83, *p* < 0.001).

## Discussion

Our present findings indicate that the administration of LFD, either alone or in combination with the tested compounds, was associated with lowered IL-1α, eotaxin-1 and leptin levels. Moreover, supplementation of LFD with P significantly increased the concentration of IL-10 vs. controls. Animals administered APO/ALA with LFD displayed a significant decrease in TNF-α concentration compared to OVA + HFD mice; those treated with ALA displayed significantly improved GSH levels. Due to the increased amount of adipose tissue, obese patients are more predisposed to systemic inflammation with higher levels of pro-inflammatory cytokines, such as interleukin 6, eotaxin-1, TNF-α, transforming growth factor beta 1 (TGF-β1) and CRP. Fatty tissue is also known to synthesize leptin. In recent years, scientists have shown that intestinal dysbiosis is associated with chronically elevated leptin levels and decreased leptin sensitivity. Leptin sensitivity may be influenced, among others, by diet, which in turn is strongly influenced by the resources of the intestinal microflora. Short-chain fatty acids (SCFAs) can activate specific signaling pathways in the host and regulate hormone secretion by affecting gut motility and fat storage in adipose tissue [20].

Both pro-inflammatory and anti-inflammatory factors may have a causal link with asthma. The presence of pro-inflammatory TNF-α increases the synthesis of IL-4 and IL-6. It may also play a role in asthmatic airway inflammation [21]. The combination of high levels of TNF-α with epithelial cell injury may lead to airway remodeling: a common feature of asthma. Several studies have found circulating concentrations of TNF-α to be elevated in obesity [22, 23]. Vieira et al. [24] report that elevated collagen levels in the lungs of OVA-challenged C57BL/6 mice were reduced by anti-TNF-α treatment. TNF-α has been found to mediate O_3_-induced lung inflammation and airway hyper-reactivity (AHR) [25, 26], and its inhibition by adiponectin can reduce AHR [27]. It was also found that adiponectin was down-regulated in a mouse model of sensitized and challenged mice [28]. Adiponectin has also been observed to inhibit TNF-α production, which might be an important target for treating obesity-related asthma [29]. Some data derived from animal models indicate that TNF-α contributes to the etiology of some obesity-related conditions, including insulin resistance [30–32].

In the present study, eotaxin-1 and IL-1α concentrations were significantly elevated in the HFD and OVA + HFD groups. In addition, eotaxin, TNF-α, and interleukins 4 and 5 levels have previously been found to be elevated in the broncho-alveolar lavage fluid (BALF) of obese mice, particularly in those with central obesity [33].

In addition, physical training combined with weight loss was found to result in significant reductions in plasma eotaxin concentrations in non-diabetic Korean women [34]. Research by Vasudevan et al. [35] confirmed that plasma eotaxin concentrations are significantly higher in obese patients than in lean controls; moreover, these values are significantly lower after weight loss, including diet-induced weight loss. An interesting study by Kouki et al. [36] found that the administration of APO reduces lung inflammation and tissue oxidative stress. The authors showed that APO also diminished LPS-induced concentrations of TNF-α and IL-1β in rat plasma. This compound restores the activity of antioxidant enzymes, such as SOD and CAT, in the lungs, thus reducing protein and lipid oxidation. In another work, OVA-challenged mice not treated with APO exhibited substantial increases in plasma cytokine concentration, particularly TNFα and IL-4 and IL-6. Nesi et al. [37] indicate that APO improves AHR, lowers the concentration of IL-4 and IL-13, and inhibits the activity of eosinophil peroxidase.

It has been reported that leptin can increase OS by inducing monocyte proliferation, resulting in increased NADPH oxidase activity. Frühbeck et al. [38] note that low adiponectin/leptin ratios exacerbate OS and inflammation.

Our present findings indicate that the administration of LFD with ALA was associated with lower concentrations of leptin. This has been confirmed by other authors [39]. Rahmanabadi et al. [40] report that oral ALA supplementation (1200 mg/12 weeks) resulted in a significant reduction in the serum leptin concentration and a noticeable elevation in the adiponectin-to-leptin ratio in patients with non-alcoholic fatty liver disease. Prieto-Hontoria et al. [41] confirm that dietary supplementation with lipoic acid decreased both circulating leptin, and adipose tissue leptin mRNA in rats, while Aslfalah et al. [42] report lower maternal circulating values of leptin in women with gestational diabetes mellitus following daily supplementation with ALA (100 mg/8 weeks). Additionally, Zhang et al. indicate that ALA may be used for the treatment of postoperative cognitive dysfunction [43]. A randomized, double-blind, placebo-controlled crossover clinical trial found that treatment with oral ALA (1200 mg/8 weeks) resulted in a significant reduction in both waist circumference and body weight [44].

However, it is possible that lipoic acid can also promote the secretion of leptin from adipocytes. Kandeil et al. [45] report that ALA treatment (100 mg daily) significantly increased serum leptin and insulin levels in comparison with an untreated diabetic group.

A large body of data exists regarding the use of probiotics in the treatment of asthma/obesity [46, 47]. Some studies have identified changes in the gut microbiota as a potential factor in the pathophysiology of obesity and asthma. Data available so far suggest that HFDs are associated with a reduction in intestinal bacterial diversity, changes in membrane integrity and the immune system, and low-intensity systemic inflammation. Many studies have highlighted the role of dysbiosis in obesity [48, 49], with some studies indicating it could be modulated by several months on an LFD [50, 51].

A limited number of studies have examined the effect of probiotic supplementation on OS and inflammation parameters in obesity-associated asthma. Shore [52] suggests that strategies (probiotics/prebiotics) that alter the gut microbiome or/and its metabolic products, such as dietary manipulations, may represent a new frontier for treating asthma in obese individuals. Our present findings also indicate a significant decrease in the levels of eotaxin, IL-1α, and leptin in the probiotic-treated group. Moreover, weight loss achieved by dieting is capable of increasing lung function in obese asthmatic patients, measured as improvements in forced expiratory volume in the first second (FEV_1_) and forced vital capacity (FVC).

Several recently published papers have examined the potential role of probiotics in the prevention or/and treatment of various diseases. Some works propose an antioxidant hypothesis to explain the prevention of cardiovascular disease using *Lactobacillus*. It has been reported that *Lactobacillus plantarum* SC4 and *L. plantarum* CAI6 may prevent cardiovascular disease in hyperlipidemic mice by Nrf2-induced anti-oxidative defense and regulation of lipid metabolism [53]. It has also been suggested that probiotic pre-treatment could successfully prevent acute pancreatitis-induced glutathione depletion and stimulate glutathione biosynthesis [54, 55].

Our present findings indicate that significantly lower concentrations of GSHt, GSH, and thiol groups were present in the lungs of asthmatic, obese, and asthmatic obese animals than in controls. In addition, the obese and asthmatic obese animals presented significantly higher GSSG levels than the controls.

Our findings indicate a decrease in –SH group content and reductions in GSHt and GSH levels in OVA/HFD-treated mice; these changes may be associated with an increase in ROS concentration in the lung homogenates [56]. Yamato et al. [57] report that the presence of fatty acids in blood plasma increases the circulating levels of OS factors in obese mice, resulting in a decrease in –SH group levels. Delwing-de Lima et al. [58] report significantly lower total sulfhydryl content in obese rats compared to those of normal weight. Several recent papers examining the use of lipoic acid have focused on its role in the treatment of sepsis, ischemic-reperfusion, neurodegenerations, diabetes, and endotoxin-induced stress [59–63].

Our data show that obese and asthmatic obese animals demonstrate significantly lower GSHt and GSH levels than controls. Insufficient concentrations of glutathione may increase the accumulation of ROS, including that of the highly reactive hydroxyl radical, which plays a key role in membrane lipid peroxidation and cellular structure damage [64].

Recently, several studies have suggested that people with asthma or obesity tend to demonstrate lower concentrations of total and reduced glutathione. Zheng et al. [65] report significantly decreased activities of antioxidant stress-related enzymes (GPx and SOD) and lower levels of GSH in the lung, heart, and kidney tissues of obese, asthmatic, and obese asthmatic rats. Ezz-Eldin et al. [66] report that asthmatic mice showed significantly lower concentrations of GSH in lung tissue compared with controls. It has also been found that mice fed an HFD were generally characterized by lower total and reduced glutathione concentrations than those fed a standard diet [67]; the obese mice also demonstrate lower GPx activity, which catalyzes the reduction of the oxidized form of glutathione to GSH. Their findings suggest that the concentration of glutathione is lowered in the myocardium of mice; hence, an HFD may increase the risk of cardiac lipotoxicity.

The GSH/GSSG ratio is considered to be a good indicator of cellular oxidative stress [68]. Lechuga-Sancho et al. [69] report that children with high BMI have a lower GSH/GSSG ratio than controls, i.e., children with normal body weight. The authors observed a significant decrease in GSHt in the obese groups compared to controls. An interesting study by Alisik et al. [70] confirms that patients with chronic inflammation and oxidative stress in the course of rheumatoid arthritis have a higher GSSG/GSH ratio than controls.

In conclusion, our results suggest that the combination of a low-fat diet with α-lipoic acid, apocynin, or probiotics may have protective and therapeutic effects against pulmonary injury. Moreover, the weight loss resulting from a low-fat diet may improve the antioxidant defense, which is regulated by GSH.

### Supplementary Information

Below is the link to the electronic supplementary material.

Supplementary file1 (DOCX 16 KB)

## Data Availability

The data used in the present study are available from the corresponding author upon reasonable request.

## Abbreviations

ALA: Alpha-lipoic acid (α-lipoic acid)
APO: Apocynin
CAT: Catalase
GPx: Glutathione peroxidase
GR: Glutathione reductase
GSH: Reduced glutathione
GSHt: Total glutathione
GSSG: Glutathione disulfide
HFD: High-fat diet
LFD: Low-fat diet
OS: Oxidative stress
OVA: Ovalbumin
P: Probiotic
ROS: Reactive oxygen species
SFD: Standard fat diet
SOD: Superoxide dismutase
