# Supplementary material for: Alpha-lipoic acid, apocynin or probiotics influence glutathione status and selected inflammatory parameters in C57/BL6 mice when combined with a low-fat diet
Source: Pharmacol Rep. 2023 Sep 21;75(5):1166–76. doi: 10.1007/s43440-023-00527-8 (PMC10539412; doi:10.1007/s43440-023-00527-8)
Supplement: Supplementary file 1 — Supplementary file1 (DOCX 16 KB) [file 43440_2023_527_MOESM1_ESM.docx]

***Biochemical determinations - Assay protocols***

*Measurement of eotaxin-1 (CCL11) concentration in the mice plasma*

Eotaxin-1 concentration was measured using Mouse CC`L11/Eotaxin Quantikine ELISA Kits (Item No. MME00), manufactured by R&D Systems Inc. (Minneapolis, MN, USA).

Assay protocol

After preparation of Eotaxin Standard wells, each well on the plate was filled with 50 µl of Assay Diluent RD1-21 and 50 μl of standard, control or plasma. Following this, the plate was covered and incubated for 120 minutes at room temperature, and then washed five times. Following this, 100 μl of Mouse Eotaxin Conjugate was added to all used wells, the plate was covered and incubated for 120 minutes at room temperature. The plate was then washed another five times and 100 μl of Substrate Solution was added. After a 30 minute incubation at room temperature, the solution was finally incubated with 100 μl of Stop Solution. Absorbance was read at 450 nm.

*Measurement of IL-1α concentration in the mice plasma*

IL-1α concentration was measured using a Mouse IL1 alpha ELISA Kit (Item No. orb50041), manufactured by Biorbyt Ltd. (Cambridge, Cambs, UK).

Assay protocol

A standard curve was prepared in the following concentrations: 300 μg/ml, 150 μg/ml, 75 μg/ ml, 37.5 μg/ml, 18.8 μg/ml, 9.4 μg/ml and 4.7 μg/ml. Following this, 100 μl of standard or samples were added to the wells and the plate was incubated at 37°C for 90 minutes. The well contents were then removed and 100 μl of biotinylated anti-mouse IL-1α antibody working solution added to all used wells. Following this, the plate was covered and incubated for one hour at 37^o^C. The plate was then washed three times with 0.01M TBS and 100 μl of prepared ABC working solution was added into each well. After a 30-minute incubation at 37°C, the plate was washed five times and 90 μl of prepared TMB color developing agent was added to all used wells, the plate was covered and incubated for 14-18 minutes at 37°C. Finally, 100 μl of prepared TMB stop solution was added. Absorbance was read at 450 nm.

*Measurement of IL-10 concentration in the mice plasma*

IL-10 concentration was measured using a Mouse IL-10 ELISA Kit (Item No. orb50056), manufactured by Biorbyt Ltd. (Cambridge, Cambs, UK).

Assay protocol

A standard curve was prepared in the following concentrations: 1000 pg/ml, 500 pg/ml, 250 pg/ml, 125 pg/ml, 62.5 pg/ml, 31.25 pg/ml and 15.625 pg/ml. After the standards were prepared, the wells on the plate were filled with 100 µl of each standard (Mouse IL-10 standard solutions), control or sample (plasma). Following this, the plate was covered and incubated for 90 minutes at 37°C. Next, 100 µl of biotinylated anti-Mouse IL-10 antibody working solution was added. After 60-minute incubation at 37°C, the plate was washed three times with 0.01 M TBS, and 100 µl of ABC working solution was added. After a 30-minute incubation at 37°C, the plate was washed five times with 0.01 M TBS and 90μl of prepared TMB color developing agent was added to each well. After another 30-minute incubation at 37°C, 100 µl of prepared TMB stop solution was added into each well. Absorbance was read at 450 nm.

*Measurement of leptin concentration in mouse plasma*

Leptin concentration was measured using a Mouse Leptin ELISA Kit (Item No. orb385387), manufactured by Biorbyt Ltd. (Cambridge, Cambs, UK).

Assay protocol

A standard curve was prepared in the following concentrations: 8000 pg/ml, 4000 pg/ml, 2000 pg/ml, 1000 pg/ml, 500 pg/ml, 250 pg/ml and 125 pg/ml. After preparation of standards, each well on the plate was filled with 100 µl of each standard (Mouse Leptin Standard solutions) or sample (plasma). Following this, the plate was covered and incubated for 12 hours at 4°C and washed three times with Wash Buffer Working Solution. Next, 100 μl of Biotin-Labeled Detection Antibody Working Solution was added; the plate was incubated for 60 minutes at 37°C, washed three times, and 100 μl of Streptavidin-HRP Working Solution was added. After a 45-minute incubation at 37°C, the plate was washed five times. Following this, 100 μl of TMB Substrate Solution was added into each well, the plate was incubated for 30 minutes at 37°C (in dark) and then 100 µl of Stop Solution was added into each well. Absorbance was read at 450 nm.

*Measurement of* *TNF-α concentrations in mouse plasma*

TNF-α concentration was measured using Mouse TNF-α Immunoassay Quantikine ELISA Kit (Item No. MTA00B), manufactured by R&D Systems Inc. (Minneapolis, MN, USA).

Assay protocol

A standard curve was prepared in the following concentrations: 700 pg/ml, 350 pg/ml, 175 pg/ml, 87.5 pg/ml, 43.8 pg/ml, 21.9 pg/ml and 10.9 pg/ml. Subsequently, 50 μl of Assay Diluent RD1-63 was added to each well. After preparation of standards, each well on the plate was filled with 50 µl of each standard (Mouse TNF-α Standard solutions) or control or sample (plasma). The plate was then incubated at room temperature for two hours. Following this, the plate was washed five times (after removing the wells). Next, 100 µl of Mouse TNF-α Conjugate was added. The plate was incubated at room temperature for two- hours, and washed five times. Next, 100 μl of Substrate Solution was added. After a 30-minute incubation in the dark, 100 µl of Stop Solution was added into each well. Absorbance was read at 450 nm.
